# Supplementary material for: Identification of hot spring Obelisk-like RNA replicons and expanded diversity of the Obelisk superfamily
Source: Nat Commun. 2026 Apr 20;17:3041. doi: 10.1038/s41467-026-71096-6 (PMC13096302; doi:10.1038/s41467-026-71096-6)
Supplement: Supplementary file 1 — Supplementary Information [file 41467_2026_71096_MOESM1_ESM.pdf]

FLDS experimental methods

Overview

Two versions of Fragmented and primer-Ligated dsRNA Sequencing (FLDS) were used in this study:

- FLDS version 2 [1]:
  Used for environmental dsRNA metatranscriptomes from hot spring samples.

- FLDS version 3 [2]:
  Used for plant leaf (CSVd) dsRNA-seq validation.

Both versions share the core workflow: dsRNA enrichment → mechanical fragmentation → adaptor ligation → reverse transcription → PCR amplification → NGS library preparation.

Section 1 — FLDS version 2 (Hot spring dsRNA metatranscriptomes)

FLDS version 2 was used to construct dsRNA-derived metatranscriptomes from hot spring samples. The protocol follows Urayama et al. (2018) [1] with minor modifications and was performed in a previous study [3].

1-1. dsRNA extraction and purification

Frozen stored (-80˚C) microbial cells on cellulose acetate filter (pore size of 0.2 μm) was flash-frozen in liquid nitrogen and pulverized in a 60-mm mortar while intermittently adding liquid nitrogen to maintain frozen conditions. Total nucleic acids were extracted using 0.5 ml of 2×STE extraction buffer (20 mM Tris–HCl [pH 6.8], 200 mM NaCl, 2 mM EDTA, 1% SDS, 0.1% [v/v] β-mercaptoethanol). After two times of phenol/chloroform/isoamyl alcohol (25:24:1) (pH 5.2, Nacalai Tesque, Kyoto, Japan) extraction, dsRNA was enriched using a cellulose column affinity chromatography (Cellulose D; ADVANTEC, Tokyo, Japan) under 1×STE buffer containing 16% ethanol, and then eluted in 1× STE buffer without ethanol [4]. The elutant was further purified with microspin cellulose columns using the same binding buffer, and dsRNA was eluted with 0.1 ml of Nuclease Free Water (NFW). Residual DNA and ssRNA were removed in a 130 μl reaction volume with 2 U of amplification-grade DNase I (Thermo Fisher Scientific, Waltham, MA, USA) and 200 U of S1 Nuclease (Takara Bio, Kusatsu, Japan) in 62 mM CH_3COONa, 10 mM $MgCl_2$, 2 mM $ZnSO_4$, and 209 mM NaCl at 37 °C for 2 h.

1-2. dsRNA fragmentation

The enzyme-treated dsRNA solution (130 μl reaction mixture) was directly subjected to ultrasound fragmentation at 4°C in Snap-Cap microTUBEs using a Covaris S220 (Woburn, MA, USA). The

fragmentation conditions were as follows: run time 35 s, peak power 140.0 W, duty factor 2.0%, and 200 cycles/burst. Then, dsRNA fragments were purified using Zymo Clean Gel RNA Recovery Kit (Zymo Research Corp., Irvine, CA, USA).

1-3. Adaptor ligation

The U2 adaptor (5′-pGAC GTA AGA ACG TCG CAC CA-p-3′) [1] was ligated to the 3′end of fragmented dsRNA in 50 mM HEPES/NaOH, pH 8.0, 18 mM MgCl2, 0.01% BSA, 1 mM ATP, 3 mM DTT, 10% DMSO, 20% polyethylene glycol 6,000, and 30 U T4 RNA ligase (Takara Bio) in a final volume of 30 μl. Incubated at 37°C for 16 h. Then, dsRNAs were purified using MinElute Gel Extraction Kit (QIAGEN, Hilden, Germany) and eluted with NFW.

1-4. Reverse transcription and cDNA amplification

U2-comp primer (0.5 μL, 5′-TGG TGC GAC GTT CTT ACG TC-3′) [1] was added. Samples were denatured at 95°C for 3 min and cooled on ice for 2 min.cDNA was synthesized using SMARTer RACE 5′/3′ Kit (Takara Bio). Residual RNA was digested with RNase H (Takara Bio) at 37°C for 10 min. cDNA was amplified by PCR with KOD-Plus-Neo (Toyobo, Osaka, Japan) using 0.25 μM U2-comp and 0.25 μM UPM primer (provided by the SMARTer RACE 5′/3′ Kit). The PCR cycles were as follows: 96°C for 2 min and 30–35 cycles of 98°C for 10 s, 60°C for 15 s, and 68°C for 2 min. Short cDNA and primer dimers were removed using AMPure XP beads (Beckman Coulter, Brea, CA, USA) at 0.8× the volume.

1-5. Library preparation and sequencing

cDNA was fragmented with Covaris S220 (175 W, 5% duty, 55 s, 200 cycles/burst). Libraries were constructed using KAPA Hyper Prep Kit with Illumina-compatible single-index adapters (Roche Sequencing Solutions, Wilmington, MA, USA)) according to the manufacturer's instructions. Library cleanup was performed twice using AMPure XP beads at 0.8× the volume. Quality was evaluated using Agilent Bioanalyzer 2100 (Agilent Technologies, Santa Clara, CA, USA). Sequencing was conducted on Illumina platform. Sequencing was performed on an Illumina MiSeq platform (Illumina, San Diego, CA, USA) in paired-end mode (300 × 2) with approximately 30% PhiX spike-in, yielding approximately 0.2–1.4 million paired-end reads per library.

Section 2 — FLDS version 3 (For CSVd plant leaf samples)

FLDS ver. 3 improves ligation efficiency, reduces adaptor dimers, and enables cDNA library construction and subsequent sequencing from subnanogram amounts of dsRNA (Hirai et al., 2021).

2-1. dsRNA extraction and purification

Plant leaves were flash-frozen and pulverized. Total nucleic acids were extracted with the same buffer as A1.

2-2. dsRNA fragmentation
Same with A2.

2-3. Optimized U2 adaptor ligation (ver.3)
The U2-DNA-5P3H adaptor (5′-pGAC GTA AGA ACG TCG CAC CA-H-3′) [2] was ligated to the 3′end of fragmented dsRNA in ligation buffer supplemented with T4 RNA ligase (Takara Bio) and 30 U T4 RNA ligase (Takara Bio) in a final volume of 30 µl and incubated at 37°C for 16 h. dsRNAs were purified using MinElute Gel Extraction Kit (QIAGEN, Hilden, Germany) and eluted with NFW.

2-4. Reverse transcription and cDNA synthesis
Same with A4.

2-5. PCR amplification
After the same procedure with A5, cDNA was amplified by PCR with KOD-Plus-Neo (Toyobo) using 0.25 µM U2-comp, 0.25 µM UPM primer (provided by the kit) and 1.5mM $MgSO_4$. The PCR cycles are as follows: 96°C for 2 min and 30–35 cycles of 98°C for 10 s, 60°C for 15 s, and 68°C for 2 min. Short cDNA and primer dimers were removed using the 0.7 × AMPure XP (Beckman Coulter, Brea, CA, USA), repeated twice.

2-6. Library preparation and sequencing
Library preparation and sequencing were performed as described in section A5 with minor modification. Library cleanup using AMPure XP beads was performed only once at 0.8× the reaction volume. Sequencing was conducted using the "Giga-read" sequencing service (Nippon Genetics, Tokyo, Japan) on an Illumina NovaSeq X Plus platform in paired-end mode (150 × 2), yielding approximately 22.5 million paired-end reads.

Tables

Table S1: Computed metrics reflecting the uniformity of read mapping across each candidate

Sample	Candidates	Normalized CV (< 0.6)	Normalized entropy (> 0.7)	Comment
**Leaves**	**NODE_115**	**0.581**	**0.899**	CSVd
	**NODE_392**	**0.327**	**0.930**	CSVd
**Hot Spring**	can01	1.140	0.483	
	can02	**0.564**	0.631	
	can03	1.629	0.451	
	can06a	1.532	0.634	
	can06b	1.370	0.629	
	can07	0.755	0.457	
	can09a	1.487	0.667	
	can09b	1.013	**0.751**	
	can11	0.693	**0.711**	
	**can13**	**0.381**	**0.766**	HsOb

Table S2: Summary of chemical and microbial composition [3].

	Sample	H4	H5	T1	T2	T3	T4	Y66	Y80	Y86	Oi	Ob
Chemical and isotopic composition	Ca^{2+} (mg/L)	181	195	238	401	265	223	195	202	185	26	113
	Mg^{2+} (mg/L)	7	7	8	16	10	10	8	8	8	4	139
	Na^{+} (mg/L)	77	80	995	81	77	62	53	53	49	22	2739
	K^{+} (mg/L)	112	108	106	132	140	128	117	118	97	9	310
	Cl^{-} (mg/L)	15	15	419	10	10	10	10	10	30	1	1671
	SO_4^{2-} (mg/L)	33	33	111	602	170	304	31	45	114	431	131
	SiO_2 (mg/L)	142	141	150	271	188	183	92	98	187	119	133
	δDH_2O (‰ SMOW)	-49.2	-48.1	-35.2	-25.1	-31	-33.5	-40.6	-18.8	-41.9	-30.6	-31.8
	$\delta^{18}O$-H_2O (‰ SMOW)	-7.1	-6.9	-3.3	-1.5	-3	-0.7	-4.7	3.8	-4.3	-4.4	-3.8
Relative abundances of rRNA reads	Eukaryota	0.59	0.19	1.64	0.99	7.31	1.51	0.11	1.53	5.16	0.75	4.02
	Sulfolobaceae	1	0.23	54.5	72.32	31.19	66.31	7.89	5.28	59.04	10.94	0
	other Archaea	1.01	0.19	43.23	26.29	17.83	30.36	7.06	15.11	20.99	4.83	0.14
	Hydrogenobaculaceae	96.57	98.9	0.13	0.12	0.27	1.61	84.24	77.54	14.47	49.84	0.01
	Aquificaceae	0	0.01	0	0	0	0	0.01	0	0	0.09	82.54
	Comamonadaceae	0.05	0	0	0	0.38	0	0.01	0	0	25.21	0
	other Bacteria	0.78	0.47	0.5	0.29	43.03	0.32	0.69	0.56	0.34	8.32	13.29

Supplementary Figures

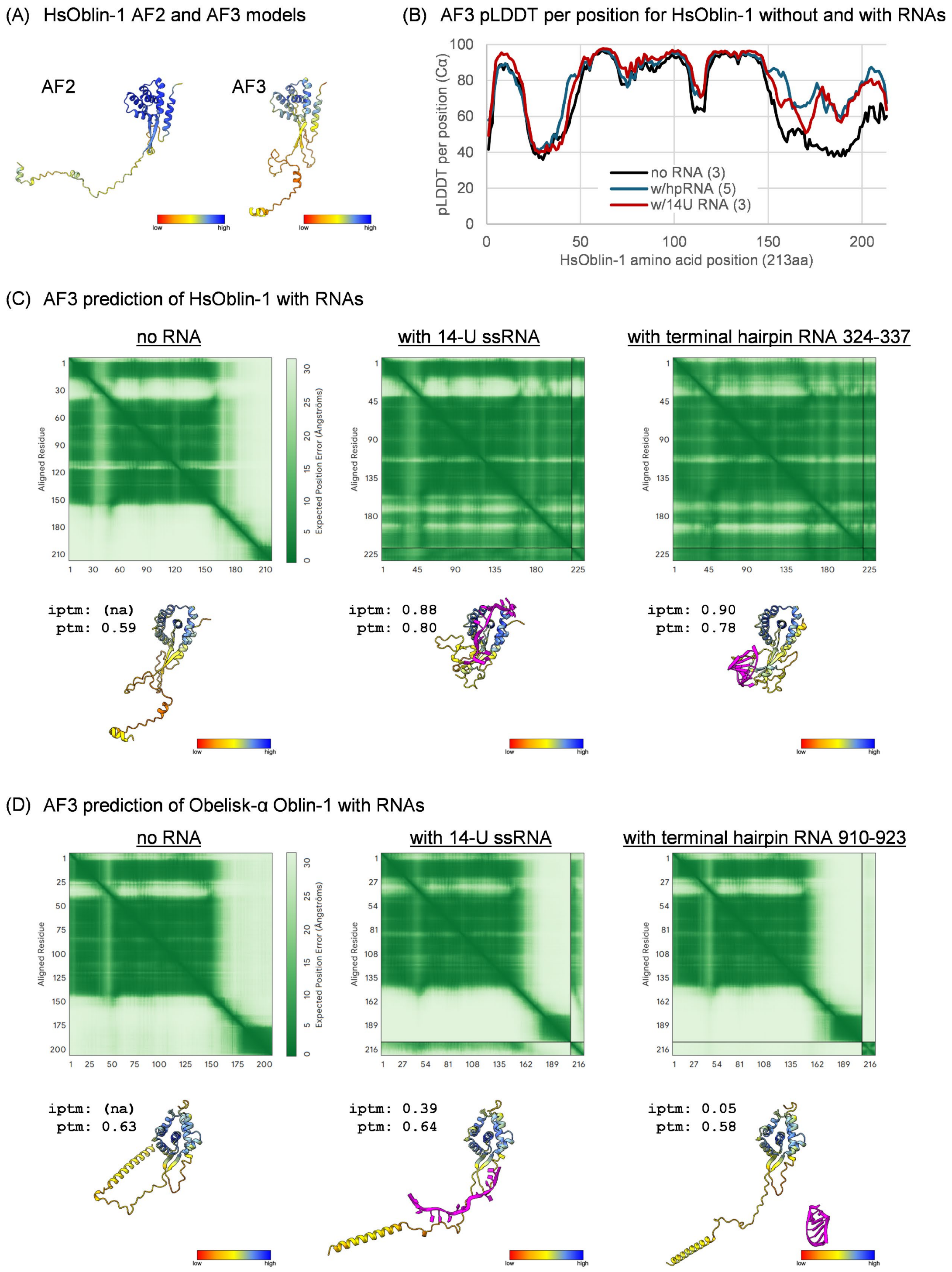

Fig. S1: Improvement of AlphaFold3 confidence metrics for HsOblin-1 structural models upon inclusion of RNA

(A) HsOblin-1-Oi structural models by AlphaFold2 (AF2) and AlphaFold3 (AF3). (B) Summary of AF3 per-residue pLDDT scores for Cα atoms from HsOblin-1-Oi predictions without and with RNA. Numbers in parentheses show technical replicates with random seeds used to average the pLDDT score. (C-D) pAE plot and predicted models of representative AF3 predictions for HsOblin-1-Oi (C) and Obelisk-α Oblin-1 (D) without and with RNAs. The protein ribbons are colored by pLDDT. RNA chains are colored purple.

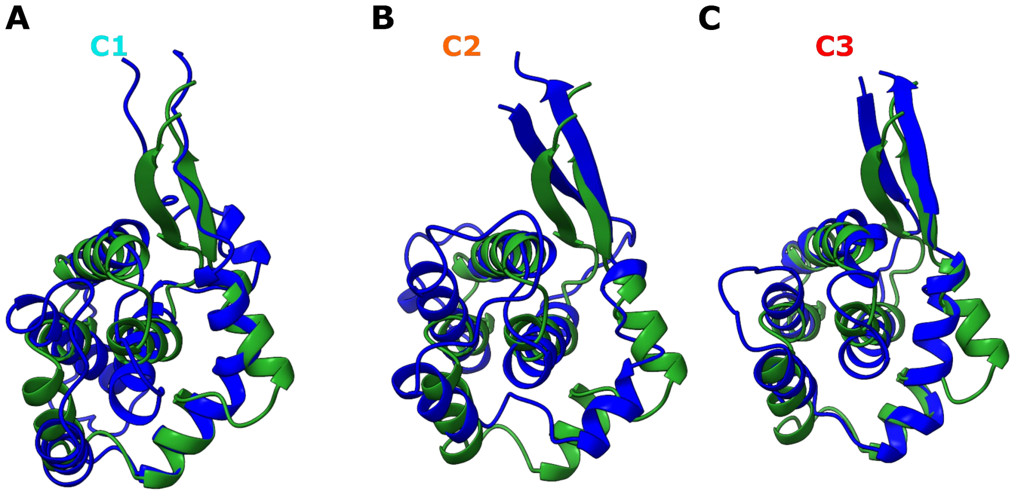

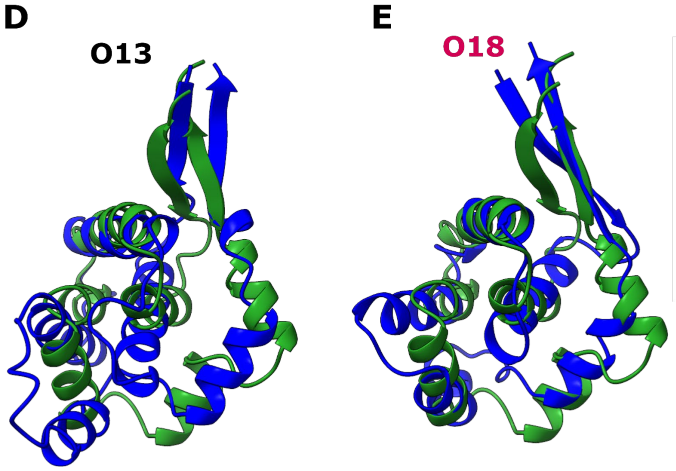

F

clade	z-score	rmsd
C1	4.8	3.2
C2	5.4	3.5
C3	7.4	2.5
O13	2.5	3.9
O18	2.6	3.9

Fig. S2: Structure comparison of different Oblin-1 subfamilies

(A-E) A representative Oblin-1 core structure (blue, globular fold flanked by the two beta-strands) of the indicated clade is superimposed to a predicted core structure of a classical Oblin-1 protein (green, globular fold flanked by the two beta-strands). (F) DALI Z-score and rmsd values of the pairwise compared core structures shown in A-E.

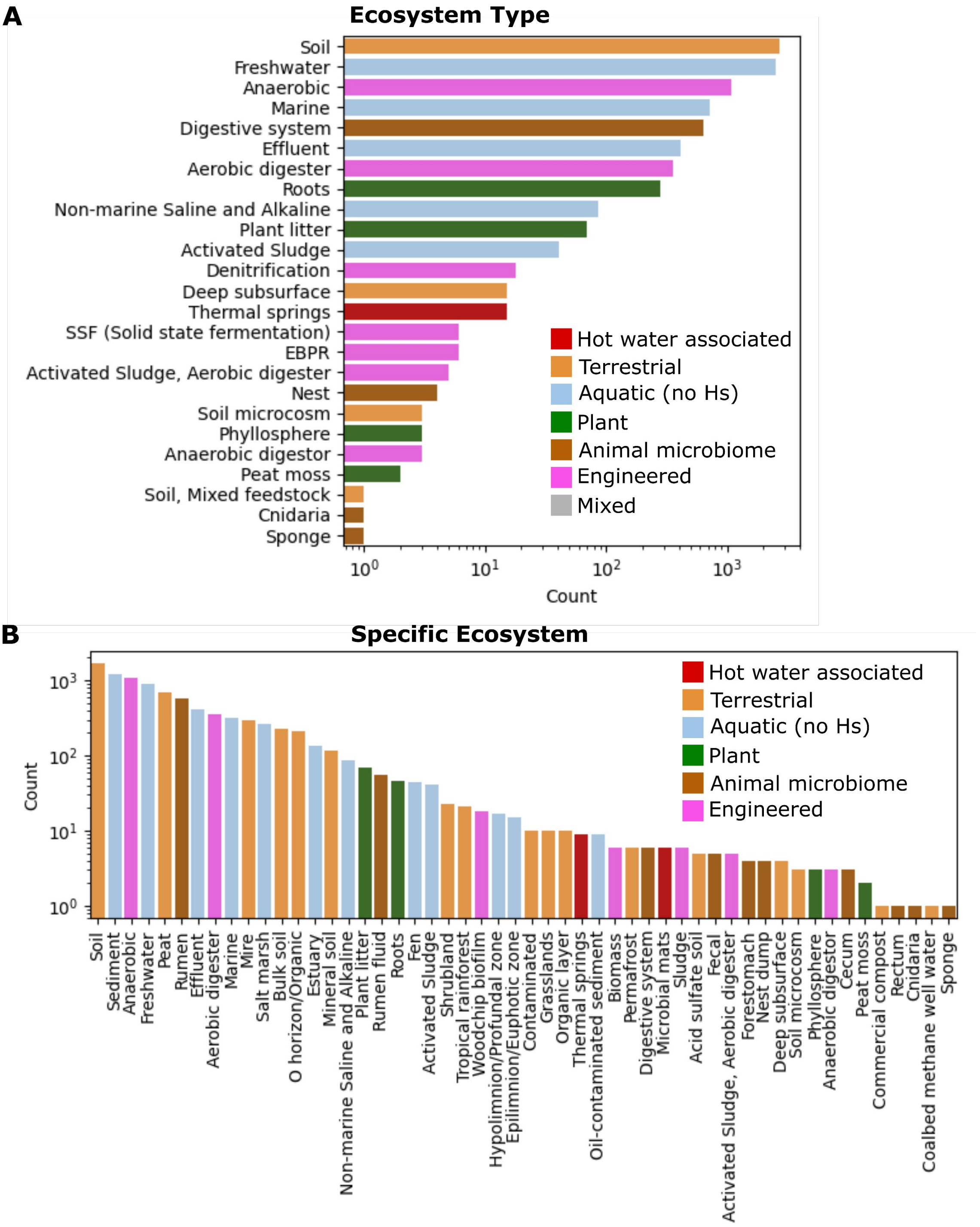

Fig. S3: Ecosystem positive for Obelisks

Ecosystems in which Oblins from all major clades have been found in. Number of metatranscriptomes with Oblins by IMG metatranscriptome annotation for ‘ecosystem type’ (A) and ‘specific ecosystem (B). Bars are colored by overarching ecosystem complementary to Fig. 4. Each Oblin and the respective metatranscriptome considered (n=9,005). Source data are provided as a Source Data file (Source_data_fig.zip).

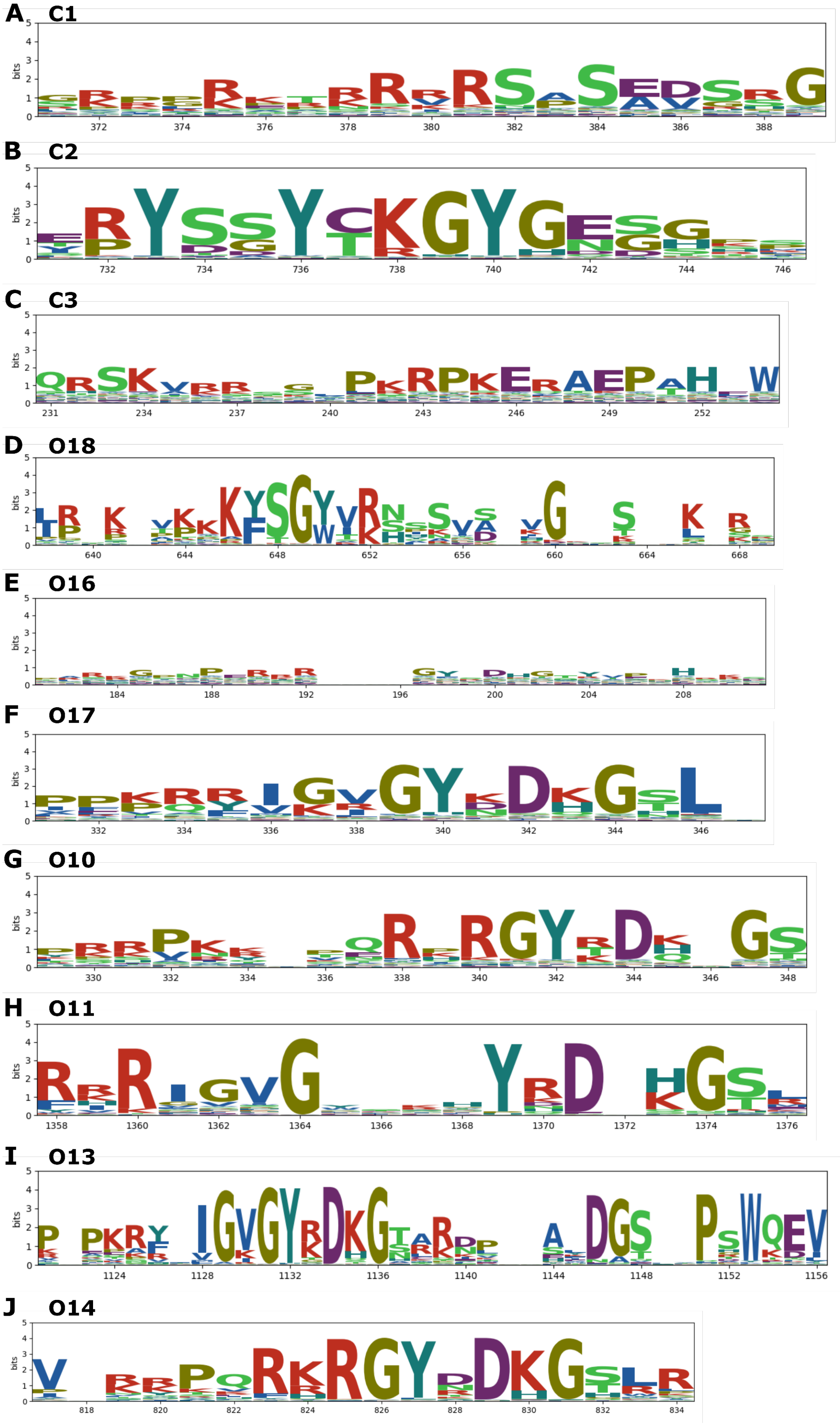
A C1
B C2
C C3
D O18
E O16
F O17
G O10
H O11
I O13
J O14
bits

Fig. S4: Sequence comparison of the 'A-domain' across Oblin-1 subfamilies

(A-J) Stacked sequence logos (information content (bits)) are shown based on the Oblin-1 alignments of the respective subfamilies. A-D show subfamilies lacking the hallmark amino acid signature GYxDxG which is present in the subfamilies shown in E-J.

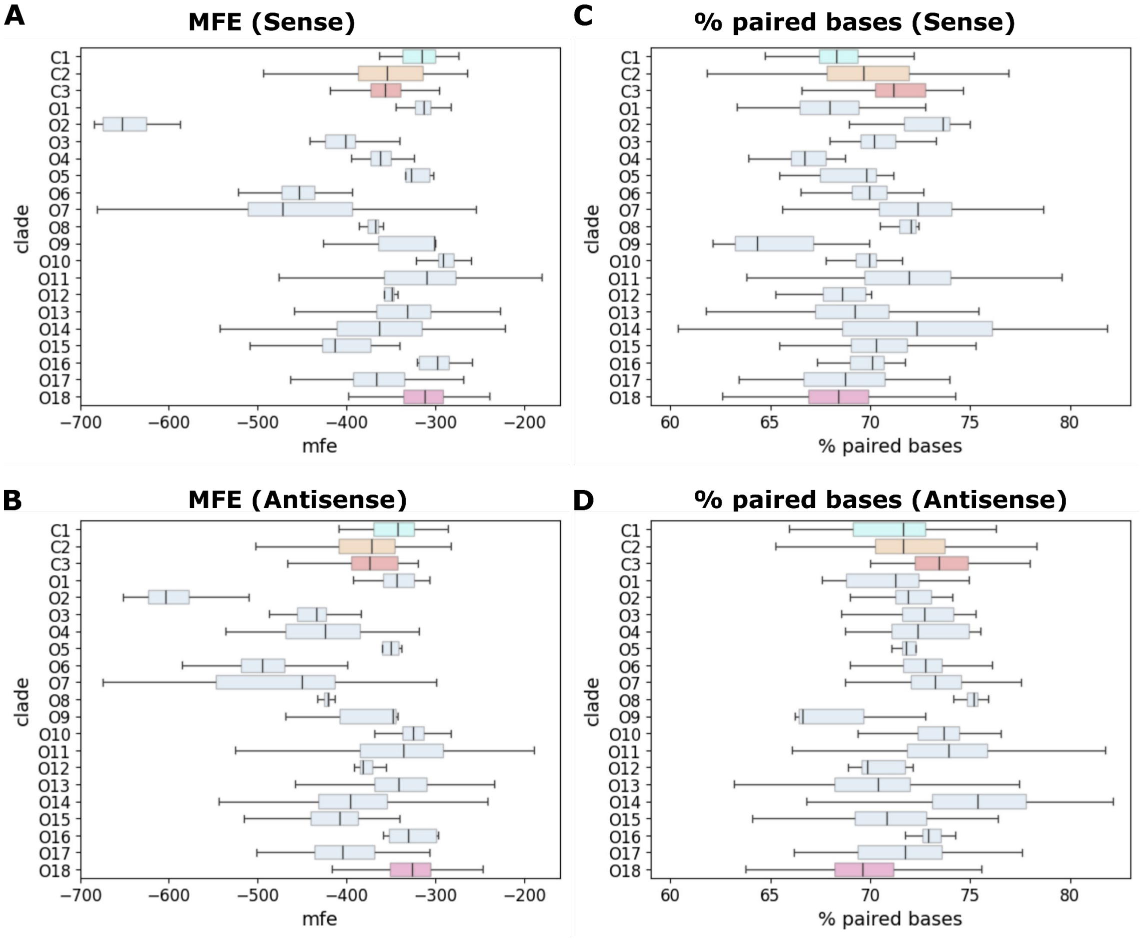

Fig. S5: Minimum free energy and percent paired bases of RNA secondary structure prediction (A,B) Minimum free energy (mfe) and (C,D) percent paired bases for Obelisk RNA secondary structure predictions by RNAfold for both the sense and the antisense genome (with respect to the Oblin-1 ORF). Boxplots show borders of first quartile, median and third quartile as box, whiskers show last datapoint within 1.5x the interquartile range. Sequences (newly discovered) (n=) per clade in each subgraph: C1: n=80; C2: n=450; C3: n=38; O1: n=42; O2: n=41; O3: n=42; O4: n=13; O5: n=9; O6: n=87; O7: n=306; O8: n=18; O9: n=3; O10: n=53; O11: n=1413; O12: n=8; O13: n=1120; O14: n=403; O15: n=118; O16: n=11; O17: n=126; O18: n=1283. Source data are provided as a Source Data file (Source_data_fig.zip).

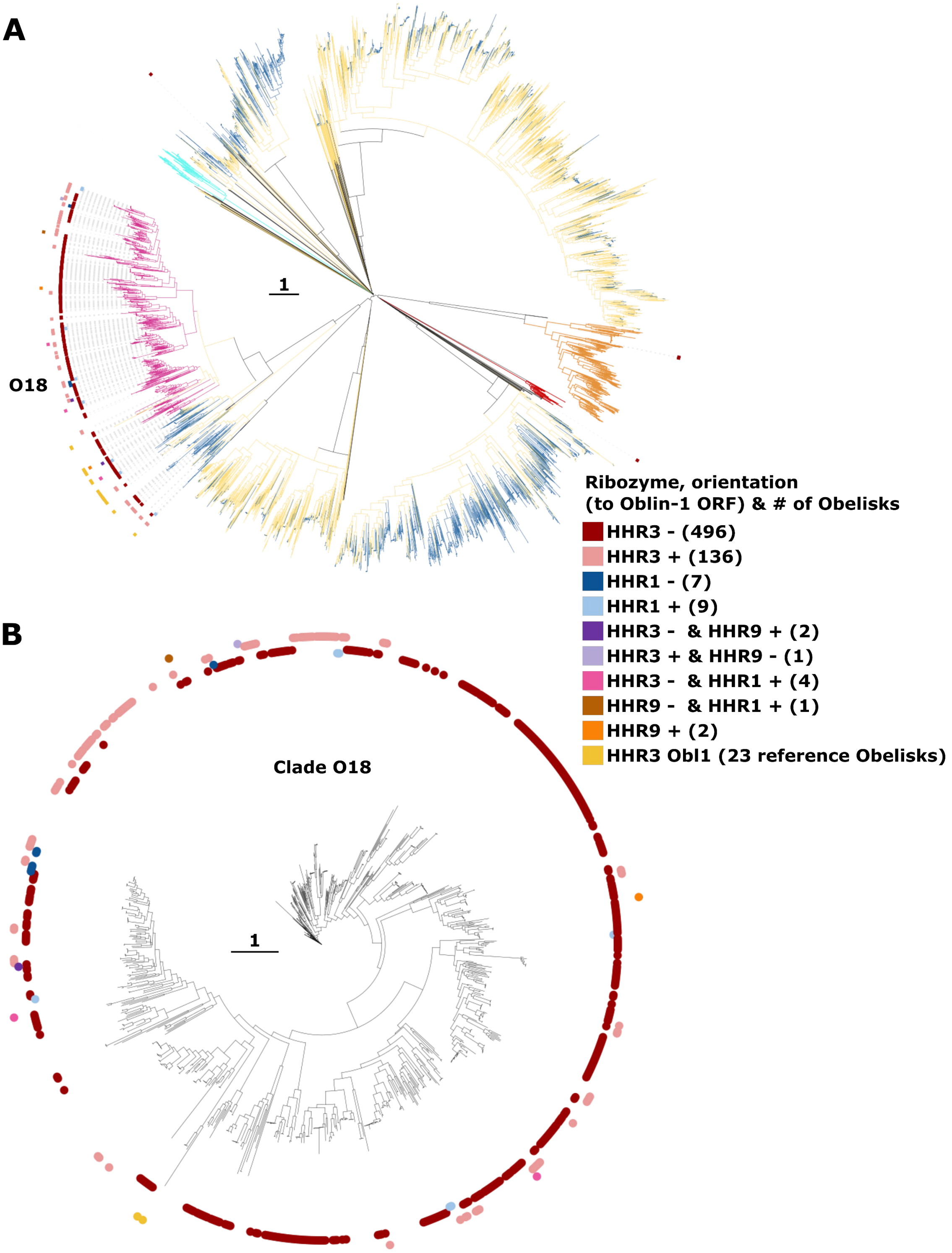

Fig. S6: Hammerhead ribozymes across Obelisks

(A) Full Oblin-1 dendrogram as in Fig.3 with Obelisks harboring a hammerhead ribozyme indicated on the rim. (B) Unrooted tree of clade O18 were the rim indicates the presence of hammerhead

ribozymes. Legend (for A,B): HHR1, 3, 9: hammerhead ribozyme 1,3 and 9, respectively; '+' or '-': ribozyme was detected on sense (+) or antisense (-) genome, with respect to the Oblin-1 ORF; HHR3 Obl1: centroid Obelisks from Zheludev et al. [5] which are reported to have a distinct HHR3; number in brackets gives the number of leaves with this type of ribozyme whereas each leaf can represent multiple Obelisks.

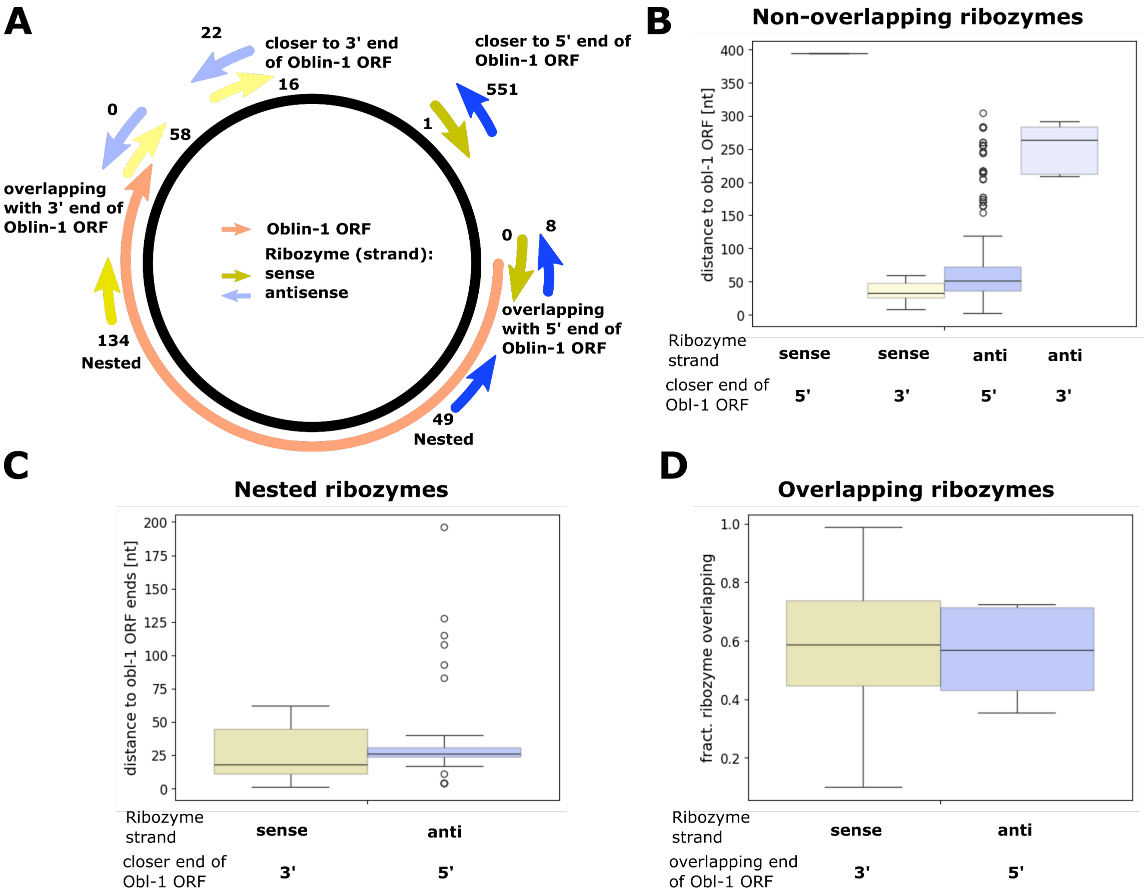

Fig. S7: Position of predicted ribozymes

(A) Schematic of the position of the predicted ribozymes and their position in relation to the Oblin-1 ORF on the cccRNA. Numbers indicate observed events of a ribozyme in that position. Arrows in blue indicate ribozymes on the antisense strand compared to the strand encoding the Oblin-1 ORF and arrows in yellow vice versa. (B) Distance of ribozyme to 5’ or 3’ end of Oblin-1 ORF (whichever is closer) if non-overlapping. Counts of Obelisks with non-overlapping ribozymes: sense-5’: n=1; sense-3’: n=16; anti-5’: n=551; anti-3’: n=22) (C) Distance of nested ribozyme to the 5’ or 3’ end of the Oblin-1 ORF (whichever is closer). Counts of Obelisks with nested ribozymes: sense-3’: n=134; anti-5’: n=49. No nested ribozymes found in the combination ‘sense and closer to 5’ end’ and ‘antisense and closer to 3’ end’. (D) Fraction of predicted ribozyme overlapping with either the 5’ or the 3’ end of the Oblin-1 ORF. Counts of Obelisks with overlapping ribozymes: sense-3’: n=58; anti-5’: n=8. (B-D) Boxplots show borders of first quartile, median and third quartile as box, whiskers show last datapoint within 1.5x the interquartile range. Dots represent outliers. Source data are provided as a Source Data file (Source_data_fig.zip).

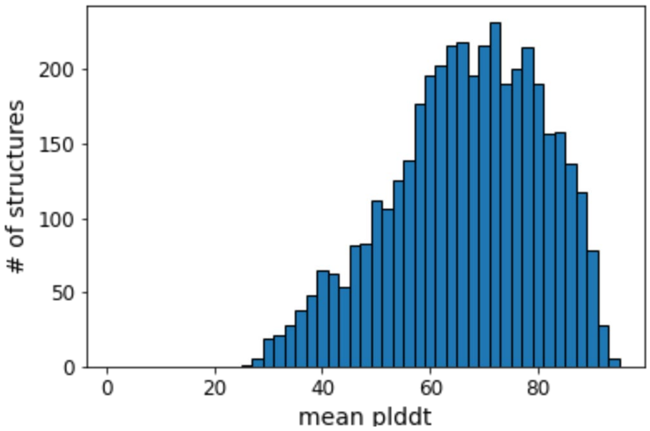

Fig. S8: Mean plddt across AlphaFold3 structure predictions for non-Oblin-1 proteins

Histogram of average plddt values for AlphaFold3 predicted structures for non-Oblin-1 proteins (n=4,124). Source data are provided as a Source Data file (Source_data_fig.zip).

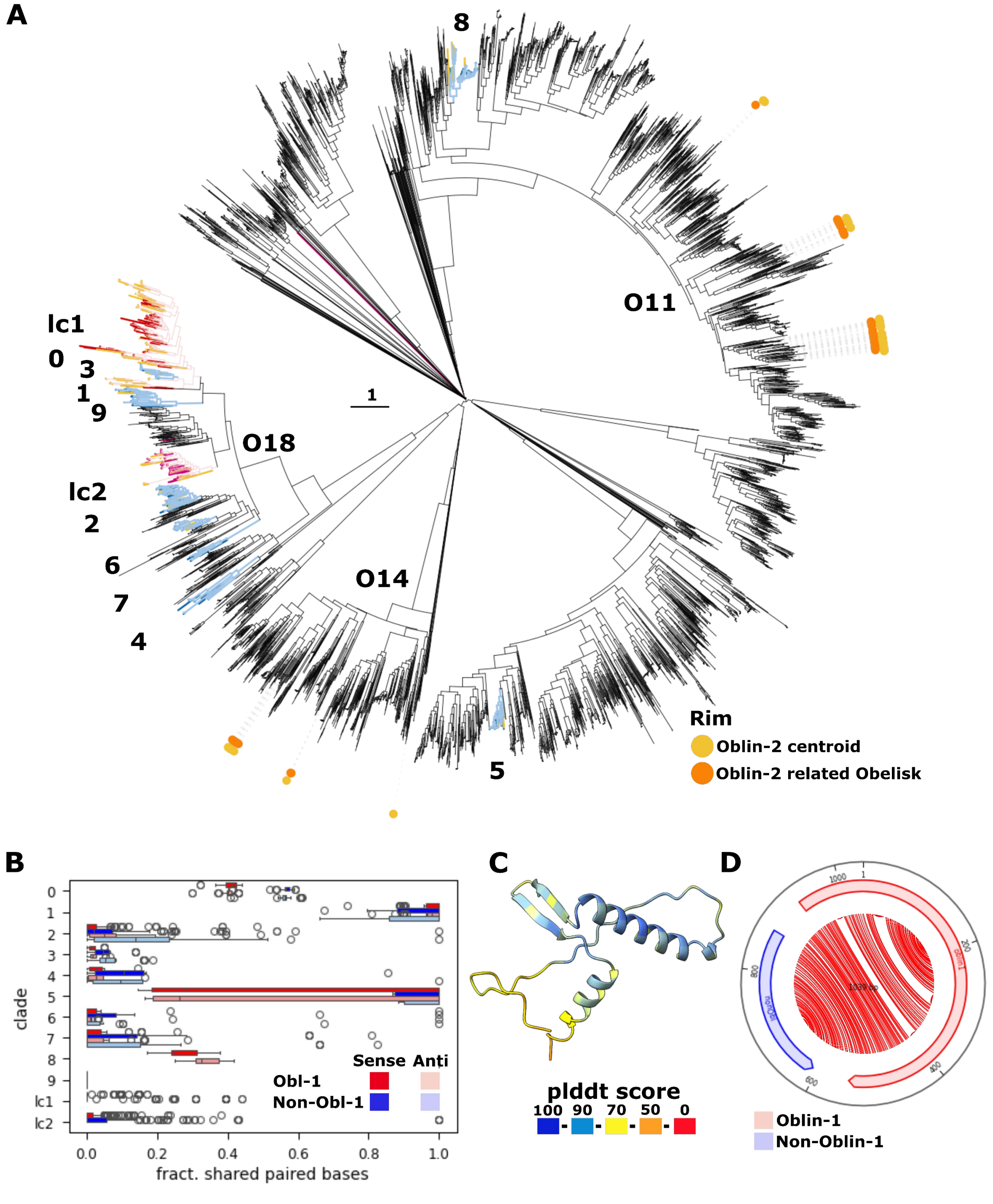

Fig. S9: Putative non-Oblin-1 proteins

(A) Clades across the Oblin-1 tree which encode for putative non-Oblin-1 proteins. More than 70% of Obelisks in blue clades harbor the respective non-Oblin-1 protein whereas coverage is lower in red clades (low coverage, lc). Yellow terminals indicate absence of the respective non-Oblin-1 protein in these clades. The presence of Oblin-2 proteins is indicated as yellow and orange dots on the outer rim (reference Obelisks and newly identified ones, respectively). (B) Fraction of paired bases between the

non-Oblin-1 (blue) and the Oblin-1 (red) ORF in the RNA secondary structure prediction of the respective Obelisks (both sense (darker color) and antisense (light color) prediction). Numbers of Obelisks per clade: '0': n=48, '1': n=62; '2': n=138; '3': n=61; '4': n=35; '5': n=49; '6': n=53; '7': n=54; '8': n=71; '9': n=70; 'lc1': n=155; 'lc2': n=172. Source data are provided as a Source Data file (Source_data_fig.zip). (C) Representative non-Oblin-1 AlphaFold3 structure prediction from group 2, colored by plddt score. (D) Obelisk map showing the non-Oblin-1 (blue) and the Oblin-1 (red) ORF and a jupiter plot of the paired bases of the structure prediction (RNAfold) of the sense genome. The Obelisks harbors the non-Oblin-1 protein from group 2 shown in C.

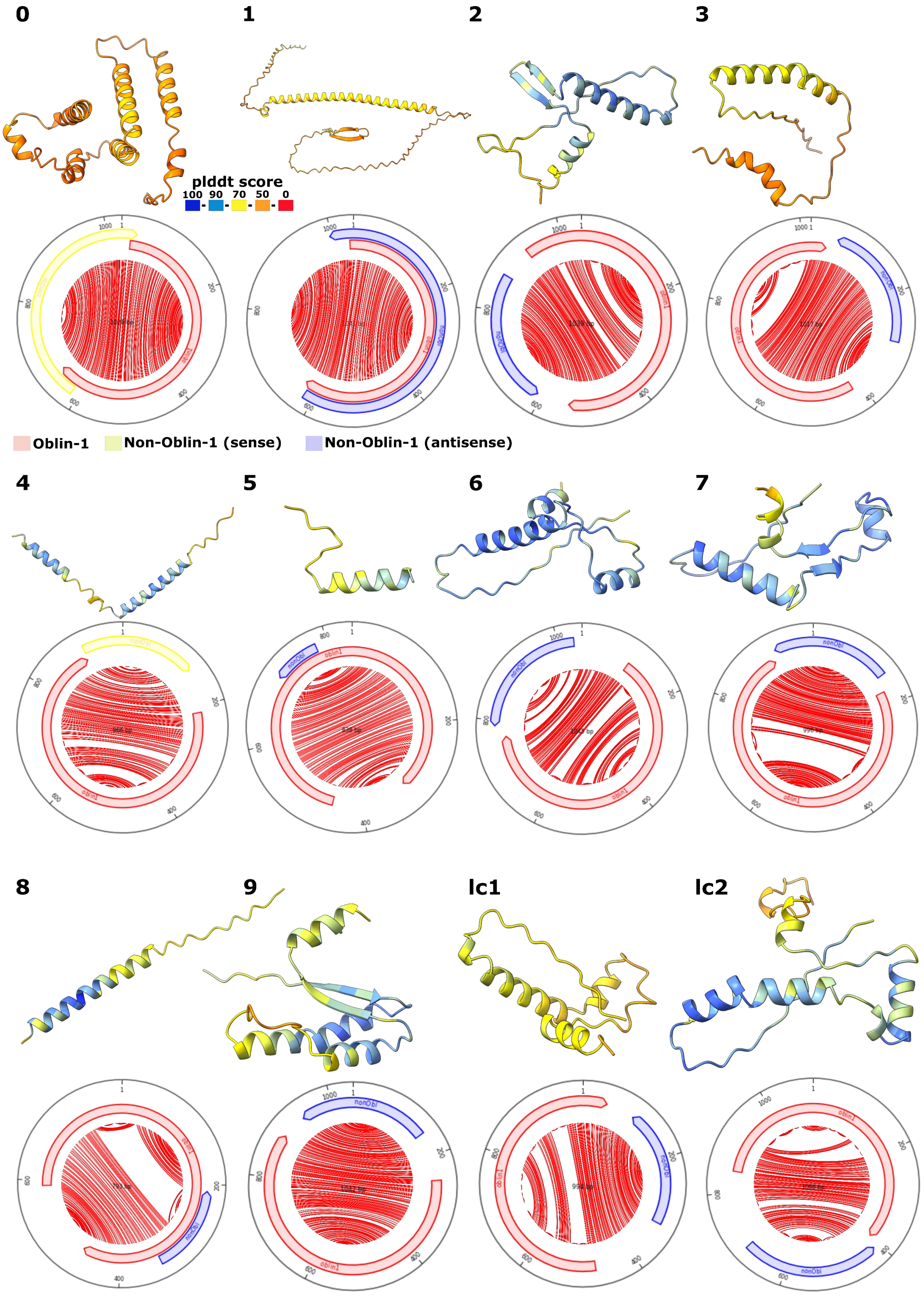
0
1
2
3
plddt score
100 90 70 50 0
Oblin-1
Non-Oblin-1 (sense)
Non-Oblin-1 (antisense)
4
5
6
7
8
9
lc1
lc2

Fig. S10: Organization of Obelisks with putative non-Oblin-1 proteins

AlphaFold3 predicted putative non-Oblin-1 structures colored by plddt score from the non-Oblin-1 groups indicated in Fig.S9 (0-9, lc1, lc2). Below each structure is the respective Obelisk map including the respective non-Oblin-1 protein (yellow, if same orientation as Oblin-1 ORF, otherwise blue) and Oblin-1 ORF (red). The jupiter plot of the paired bases of the sense genome of the respective RNA secondary structure prediction (RNAfold) is shown for each map.

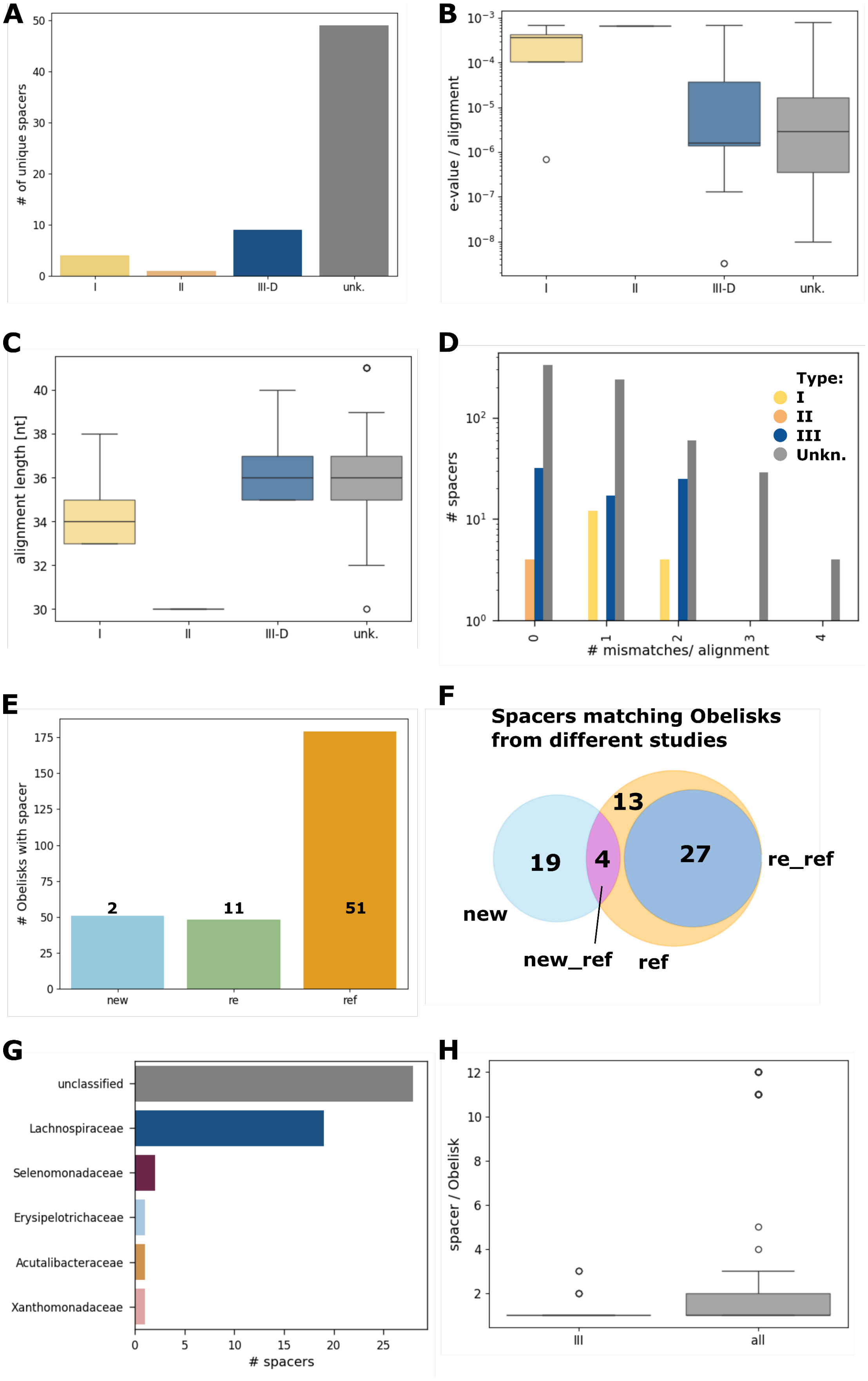
A
# of unique spacers
0
10
20
30
40
50
I
II
III-D
unk.
B
e-value / alignment
10^{-3}
10^{-4}
10^{-5}
10^{-6}
10^{-7}
10^{-8}
I
II
III-D
unk.
C
alignment length [nt]
30
32
34
36
38
40
I
II
III-D
unk.
D
spacers
10^0
10^1
10^2
Type:
I
II
III
Unkn.
0
1
2
3
4
mismatches/ alignment
E
Obelisks with spacer
0
25
50
75
100
125
150
175
2
11
51
new
re
ref
F
Spacers matching Obelisks
from different studies
19
4
13
27
new
new_ref
ref
re_ref
G
unclassified
Lachnospiraceae
Selenomonadaceae
Erysipelotrichaceae
Acutalibacteraceae
Xanthomonadaceae
0
5
10
15
20
25
spacers
H
spacer / Obelisk
2
4
6
8
10
12
III
all

Fig. S11: CRISPR spacer hits against Obelisks

Characteristics of CRISPR spacer matching Obelisk sequences. (A) Number of unique spacers associated with a CRISPR type (unkn.: unknown). Number of unique spacer per CRISPR type: ‘I’: n=4; ‘II’: n=1; ‘III-D’: n=9; ‘unkn.’: n=49. Boxplot of e-values per spacer alignment against the Obelisks (B) and spacer length (C), sorted by associated CRISPR type. (D) Number of mismatches per spacer alignment against their respective Obelisk. (B-D) Individual spacer counts per type: ‘I’: n=16; 'II': n=4; 'III-D': n=74; 'unk.': n=659) (E) Obelisks with spacers. New (n=51), re (n=48) and ref (n=179) represent Obelisks found in this study and assigned to a new 80% cluster ordinate, found in this study and assigned to a previously established 80% cluster ordinate, and Obelisks previously discovered by Zheludev et al., respectively. (F) Venn diagram of unique spacers (n=63) matching either new or reference Obelisks (as in E). (G) Spacers (n=52; 0-1 mismatches) assigned to a particular bacterial family. (H) Boxplot showing the number of spacers per Obelisk, either all spacers (n=214) or associated with CRISPR type III systems (n=64). (B, C, H) Boxplots show borders of first quartile, median and third quartile as box, whiskers show last datapoint within 1.5x the interquartile range. Dots represent outliers. Source data are provided as a Source Data file (Source_data_fig.zip).

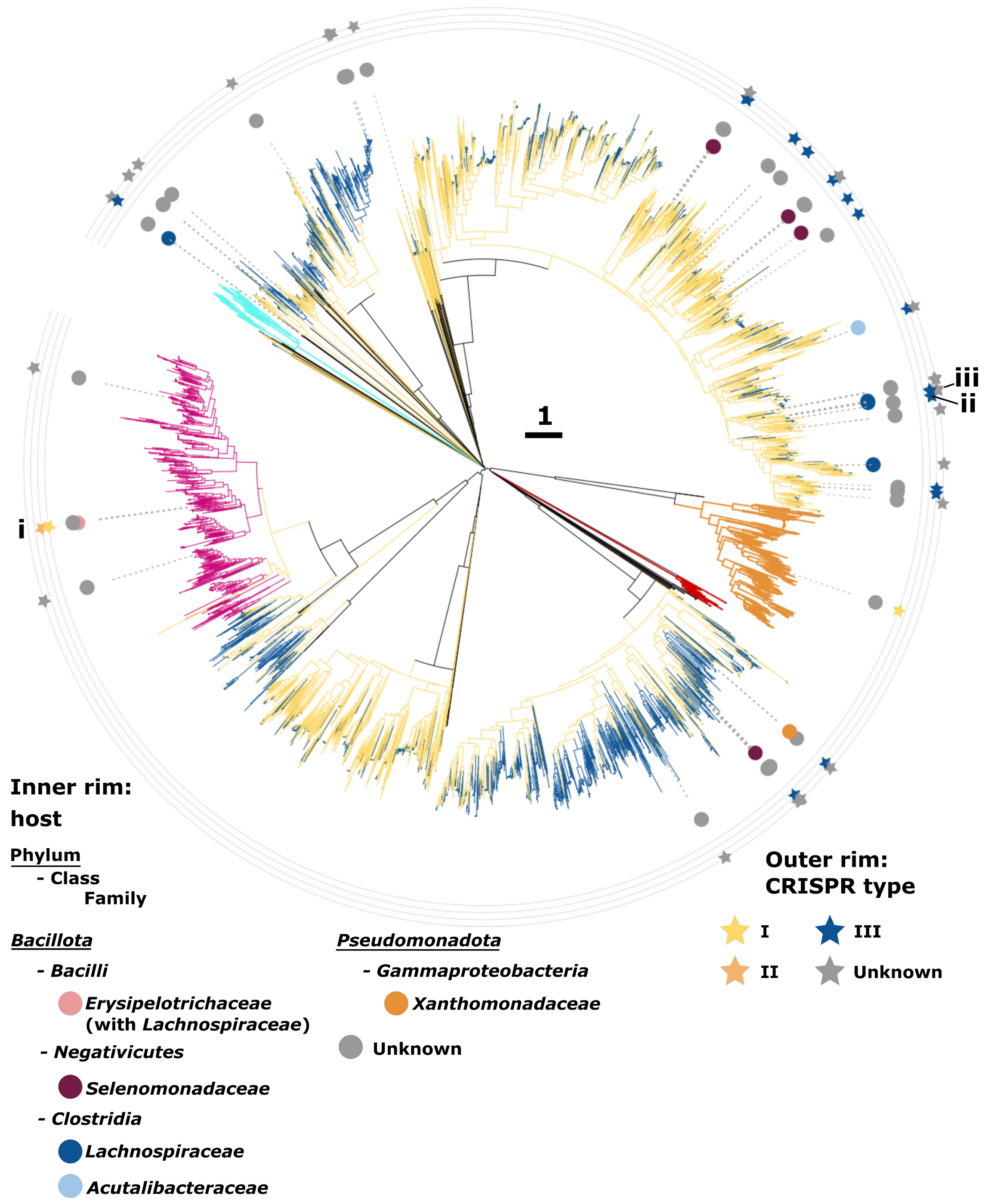

Fig. S12: Distribution of Obelisks with spacer matches along Oblin-1 tree

Obelisks with spacers were mapped to the Oblin-1 tree. The inner rim shows the associated host (Phylum, clade, and family), the other rim the respective associated CRISPR type. Roman numbers (i-iii) correspond to Obelisks shown in Fig. S13.

A B C

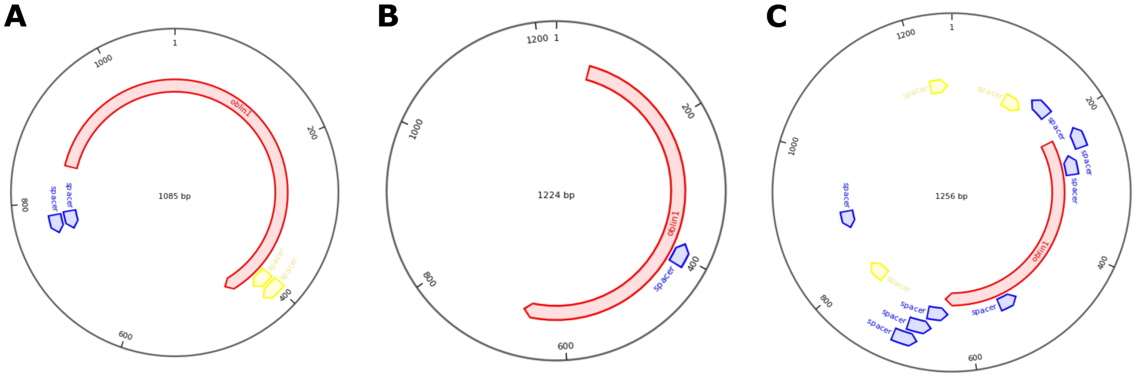

i) Obelisk_002090_000001_000001 ii) Obelisk_000324_000001_000001 iii) Obelisk_000273_000001_000013

D

	i	ii	iii
**Type**	unknown	III-D	unknown
**Spacers/Obelisk**	4	1	11
**Host family**	Erysipelotrichaceae / Lachnospiraceae	Lachnospiraceae	Lachnospiraceae
**Best spacer coveage**	7	2	3
**Mean mismatch /spacer**	0.25	1	0.55

Fig. S13: Exemplary Obelisks with at least one high confident spacer match

(A-C) Maps of 3 Obelisks with at least one high confident spacer match (0-1 mismatches, high quality flag in JGI spacer database, spacer covered by at least 2 reads). Red: Oblin-1 ORF; Yellow: spacer oriented as Oblin-1 ORF; Blue: spacer oriented reverse to Oblin-1 ORF. Spacers cover at least 10 nt non-overlapping compared to other mapped spacers. (D) Table summarizing spacer characteristics of the Obelisks in A-C. Columns: roman numbering as for maps in A-C. Rows: Type: CIRSPR type; Best spacer coverage: how many reads mapped to the spacer in the original sequencing project. Information provided by the JGI spacer db. Highest spacer read coverage shown; Mean mismatch/spacer: average mismatch of the spacer alignments.

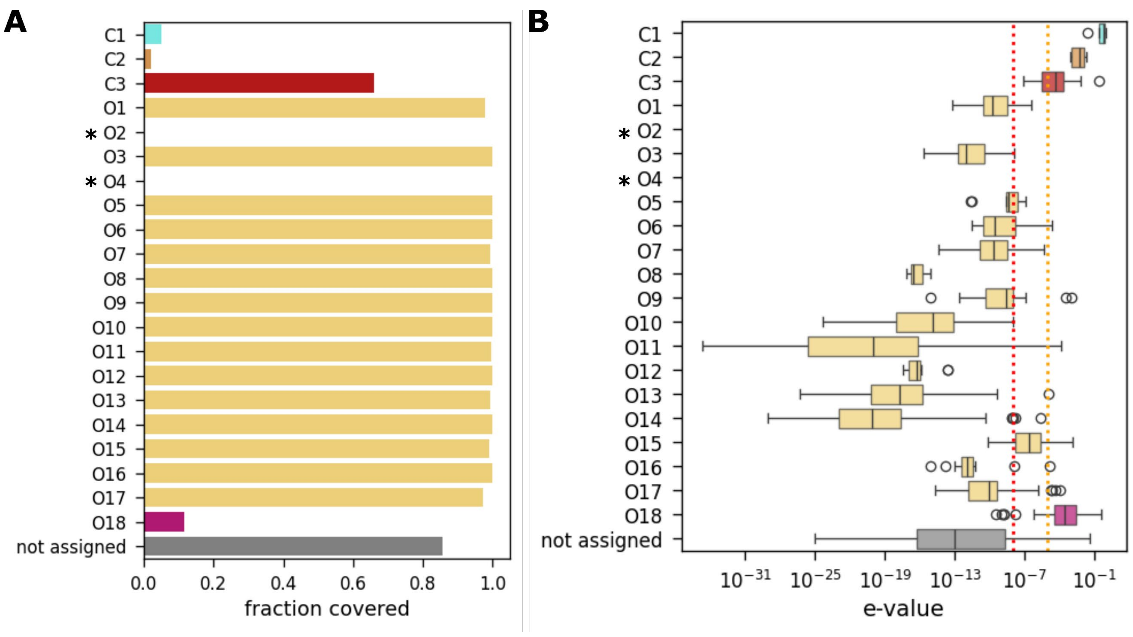

Fig. S14: Oblin-1 proteins identified with 'logan' HMM profile

Oblin-1 proteins identified in this study and from Zheludev et al. were searched with an Oblin-1 profile from a recent study mining Obelisks from the SRA ('logan' [6]). (A) reports the fraction of Oblin-1 proteins identified per clade (y axis; compare Fig.3) with an e-value <= 10. (B) Boxplot of e-values of hits per clade. Orange and red lines indicate e-value of 10-5 and 10-8, respectively. Boxplots show borders of first quartile, median, and third quartile as a box, whiskers show the last datapoint within 1.5x the interquartile range. Dots represent outliers. Colors in A and B for each clade as in Fig. 3. Asterisks indicate two small clades for which none of the members were detected by the 'logan' profile. Oblins per clade: C1: n=79; C2: n=403; C3: n=44; O1: n=49; O2: n=42; O3: n=69; O4: n=17; O5: n=10; O6: n=96; O7: n=359; O8: n=17; O9: n=16; O10: n=116; O11: n=1968; O12: n=11; O13: n=1206; O14: n=667; O15: n=124; O16: n=14; O17: n=139; O18: n=956; not assigned: n=187. Source data are provided as a Source Data file (Source_data_fig.zip).

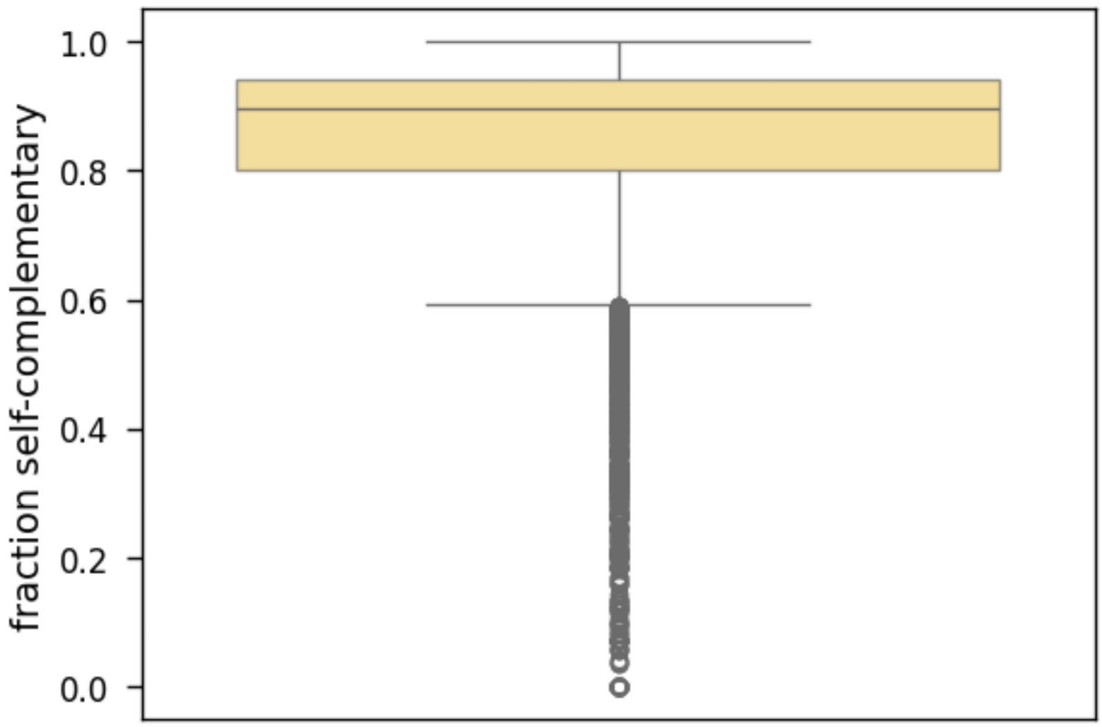

Fig. S15: Paired bases in RNA secondary structure from Oblin-1 ORF

Boxplot showing the fraction of paired bases for which both pair members reside within the Oblin-1 ORF across all paired bases for which at least one member of the pair resides in the Oblin-1 ORF. Base pairing based on the predicted RNA secondary structure of Obelisks (compare Fig. S5). Boxplots show borders of first quartile, median and third quartile as box, whiskers show last datapoint within 1.5x the interquartile range. Dots represent outliers. n=5859. Source data are provided as a Source Data file (Source_data_fig.zip).

References

1 Urayama, S. *et al.* Unveiling the RNA virosphere associated with marine microorganisms. *Mol. Ecol. Resour.* **18**, 1444-1455, doi:10.1111/1755-0998.12936 (2018).

2 Hirai, M. *et al.* RNA Viral Metagenome Analysis of Subnanogram dsRNA Using Fragmented and Primer Ligated dsRNA Sequencing (FLDS). *Microbes Environ.* **36**, ME20152, doi:10.1264/jsme2.ME20152 (2021).

3 Urayama, S. *et al.* Double-stranded RNA sequencing reveals distinct riboviruses associated with thermoacidophilic bacteria from hot springs in Japan. *Nat Microbiol* **9**, 514-523, doi:10.1038/s41564-023-01579-5 (2024).

4 Okada, R., Kiyota, E., Moriyama, H., Fukuhara, T. & Natsuaki, T. A simple and rapid method to purify viral dsRNA from plant and fungal tissue. *Journal of General Plant Pathology* **81**, 103-107, doi:10.1007/s10327-014-0575-6 (2015).

5 Zheludev, I. N. *et al.* Viroid-like colonists of human microbiomes. *Cell* **187**, 6521-6536 e6518, doi:10.1016/j.cell.2024.09.033 (2024).

6 Chikhi, R. *et al.* Logan: planetary-scale genome assembly surveys life's diversity. *bioRxiv*, 2024.2007. 2030.605881 (2025).
