# Supplementary material for: Identification of hot spring Obelisk-like RNA replicons and expanded diversity of the Obelisk superfamily
Source: Nat Commun. 2026 Apr 20;17:3041. doi: 10.1038/s41467-026-71096-6 (PMC13096302; doi:10.1038/s41467-026-71096-6)
Supplement: Supplementary file 2 — Description of Additional Supplementary Files [file 41467_2026_71096_MOESM2_ESM.pdf]

Description of Additional Supplementary Files

Supplementary Data 1

List of publicly available metatranscriptomic datasets screened for HsOb-Oi/H5-related sequences. This file contains 154 samples corresponding to 197 SRA sequencing runs retrieved from the NCBI Sequence Read Archive (SRA) and screened by BLASTN and BLASTX for homologs of HsOb-Oi and HsOb-H5.
